# Synthesis of High-Aspect-Ratio Nickel Nanowires by Dropping Method

**DOI:** 10.1186/s11671-016-1330-z

**Published:** 2016-03-01

**Authors:** Jiaqi Zhang, Wenfeng Xiang, Yuan Liu, Minghao Hu, Kun Zhao

**Affiliations:** State Key Laboratory of Heavy Oil Processing, China University of Petroleum, Beijing, 102249 China; Beijing Key Laboratory of Optical Detection Technology for Oil and Gas, China University of Petroleum, Beijing, 102249 China; College of Science, China University of Petroleum, Beijing, 102249 China

**Keywords:** Ni nanowires, Dropping method, Diameter and surface roughness, Nanocrystalline materials, Magnetic materials, 81.07.Gf, 75.50.Tt, 75.75.Cd

## Abstract

A facile and high-yield route, dropping method, has been used to synthesize Ni nanowires (NWs) with a high aspect ratio. Compared to the conventional chemical reduction method, the diameter of Ni NWs prepared by the dropping method distinctively decreased and the surface roughness was improved. After optimizing the process parameters such as the Ni ion concentration and volume of the dropped NiCl_2_·6H_2_O solution, the diameter and aspect ratio of the NWs are 70 nm and ~600, respectively. The possible synthesized process of the dropping method was discussed. This work presents a preferred approach to fabricate high-quality one-dimensional magnetic materials which have potential applications in electrochemical devices, magnetic sensors, and catalytic agents.

## Background

Over the past decades, controlled growth of nanostructure magnetic materials has been attracting considerable attention owing to their size- or shape-dependent properties [1, 2]. As a type of important magnetic material, nickel nanowires (NWs) possess unique magnetic and catalytic properties, and it has been synthesized and studied because of their potential applications in catalytic [3], microwave-absorbing materials [4], sensors [5], and memory devices [6]. To improve the performance of the materials and achieve its full potential in these applications, the preparation of Ni NWs with well-controlled size and surface roughness is critical.

The general methods for preparing Ni NWs usually require surfactants or additional template assistance, which are efficient in controlling the crystal growth and size of NWs [7, 8]. Unfortunately, these methods possibly lead to complicated fabrication processes, low cost efficiency, and low yield, which further limit the potential application. In recent years, synthesis of NWs by chemical reduction route is the preferred approach as it is low-costing, environmentally friendly, safe, and feasible for large-scale production. Liu, et al. [9] synthesized nickel NWs by assembly of small nanoparticles. Tang et al. [10] prepared the Ni NWs with chain morphology using the microwave-assisted method. However, to control the size and morphology of NWs synthesized by this method is still a challenge; it therefore becomes an interesting research field to precisely control the size and shape of these materials.

In this work, we propose a modified chemical reduction process, dropping method, to synthesize Ni NWs. In this method, the diameter of Ni NWs was effectively controlled, and the aspect ratio of NWs has been improved under a very low magnetic field (~0.05 T). Then, a possible synthesized process has been described. This method may further shed light on the fabrication approach on magnetic NWs with high quality.

## Methods

During the synthesis process of Ni NWs by dropping method, 1.2 g sodium hydroxide (NaOH) was dissolved in 35 ml ethylene glycol (EG) under continuous magnetic stirring for 1 h. And then, 10 ml hydrazine hydrate (N_2_H_4_·H_2_O, 80 wt%) solution as a reducing agent was added into the NaOH and EG mixed solution with constant stirring to obtain a homogeneous solution. The as-prepared solution was placed in a magnetic field and heated to 80 °C at atmospheric pressure. The strength of the magnetic field in the container was 0.05 T generated by a NdFeB magnet. Then, 5 ml EG solution of nickel chloride hexahydrate (NiCl_2_·6H_2_O, 0.1 M) was added dropwise into the abovementioned solution. After about 10 min, a black fluffy solid product was formed and adsorbed on the inner surface of the container. The products were collected using a magnetic field and washed repeatedly with distilled water and ethanol and then dried at 60 °C for 12 h. The NWs prepared by the dropping method are named as DM-NWs. For comparison, Ni NWs were prepared by a conventional chemical reduction method [11]. In this method, a homogeneous blue solution was obtained by fully mixing all the abovementioned solutions alternately and the Ni NWs were synthesized with the same conditions of the dropping method. The NWs synthesized by the conventional method are named as CM-NWs. The size and surface morphology analyses were performed using a field emission scanning electron microscope (SEM, Hitachi SU8010, Japan). The phase structure and composition of NWs were characterized by an X-ray polycrystalline diffractometer (XRD, D8 Advance, Bruker) using Cu K*α* radiation with a graphite monochromator. The 2*θ* was scanned over the wide-angle range of 20°–90° at a rate of 1°/min to identify the structure. The detailed microstructure of the samples was studied by a transmission electron microscopy (TEM, JEM-2100, Japan).

## Results and Discussion

**Fig. 1:**
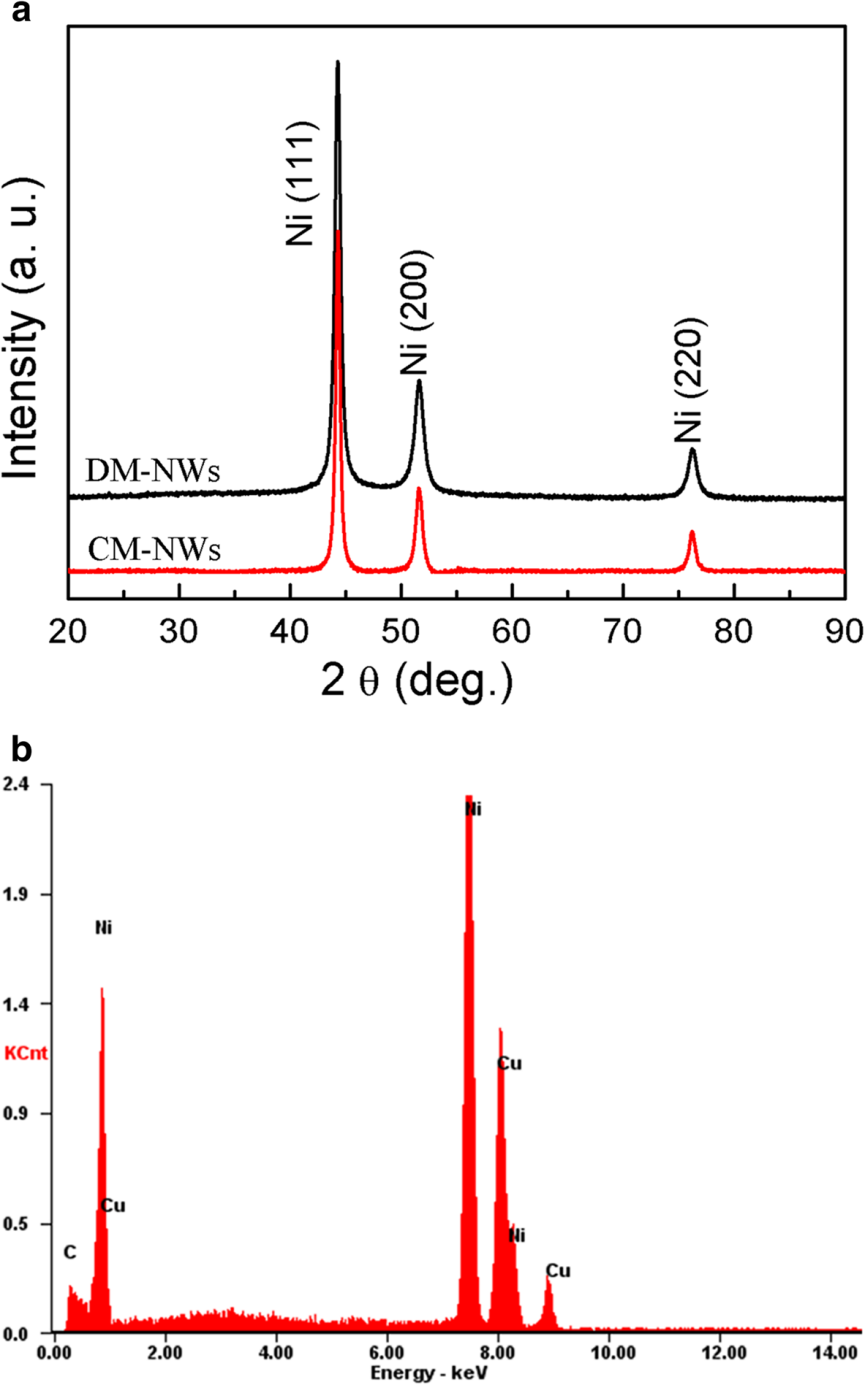
XRD patterns and EDX spectrum of Ni NWs. **a** XRD patterns of the DM-NWs and CM-NWs. **b** EDX spectrum of the DM-NWs

XRD patterns of DM-NWs and CM-NWs are shown in Fig. 1a. All the reflection peaks can be well matched with the face-centered cubic nickel (JCPDS 04-0850, space group, Fm3m (225)). The three peaks correspond to crystal planes of (111), (200), and (220) of crystalline Ni, respectively. Energy-dispersive X-ray spectrum (EDX) analysis (shown in Fig. 1b) of the DM-NWs shows that only Ni element exist in the Ni NWs (peaks of Cu and C can be ascribed to the Cu and C grid). From the XRD patterns and EDX results, no impurities such as NiO or Ni(OH)_2_ occurred, indicating that phase-pure Ni NWs were obtained via the dropping method.

**Fig. 2:**
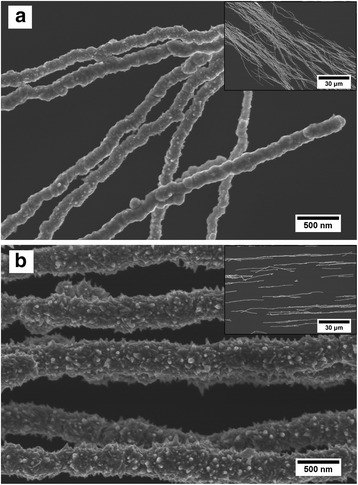
SEM images of nickel NWs. **a** SEM images of nickel NWs prepared by the dropping method. **b** SEM images of nickel NWs prepared by the conventional method

The SEM micrographs of DM-NWs and CM-NWs are shown in Fig. 2. It can be seen that the DM-NWs (Fig. 2a), with diameters of ~200 nm, consist of nanoparticles with a smooth surface. Though the CM-NWs (Fig. 2b) possess relatively uniform diameters (~450 nm), the NWs were packed with spiky thorns all over the surface. The surface thorns grow perpendicularly standing along the radial direction and present a conical construction with a length of 20–50 nm. These two kinds of NWs have a similar length as shown in the insets of Fig. 2a, b. Compared to the CM-NWs, the diameter of DM-NWs distinctively decreased without changing the NW length, i.e., the aspect ratio of DM-NWs is better than that of CM-NWs.

**Fig. 3:**
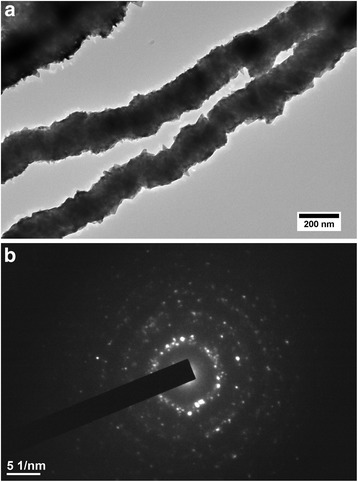
TEM micrograph and SAED pattern of Ni NWs. **a** TEM micrograph of Ni NWs synthesized by the dropping method. **b** SAED pattern of Ni NWs synthesized by the dropping method

For better understanding of the synthesized mechanism and microstructure of Ni NWs, TEM measurement was carried out as shown in Fig. 3a. It can be found that a slight contrast effect in Ni NWs was observed, indicating that the NWs are assembled by a closely aligned array of nickel nanoparticles under the magnetic field. The selected area electron diffraction (SAED) pattern (Fig. 3b) carried on a single Ni NW consists of diffraction rings, indicating that the Ni NWs have a polycrystalline structure with a FCC structure, which is in agreement with the XRD results.

**Fig. 4:**
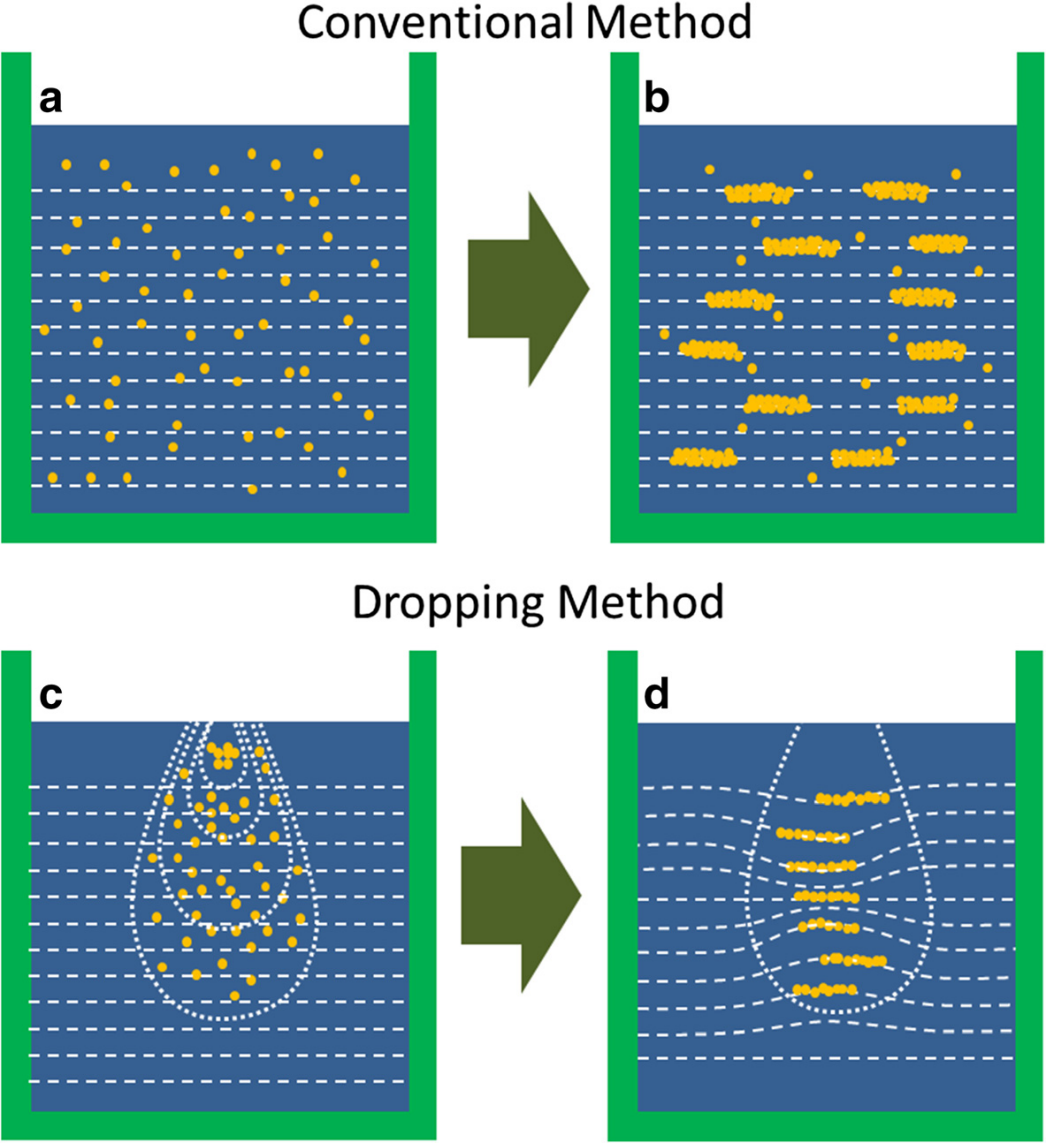
Schematic illustration of the synthesis of Ni NWs. **a**, **b** The process to synthesize CM-NWs. **c**, **d** The process to synthesize DM-NWs. In the figure, the *yellow spots* represent the Ni nanoparticles, the *dashed lines* represent the magnetic induction line, and the *drop-shaped area* bounded by the *dotted line* indicates the diffusion region of the NiCl_2_·6H_2_O solution followed by the reaction time

Many groups in the world have investigated the synthesized mechanism of CM-NWs [12–14]. For the dropping method, the synthesized mechanism is similar with the conventional reduction method. However, the synthesized process is different from each other. Figure 4a–d schematically shows the difference of the synthesized process between CM-NWs and DM-NWs. During the synthesis of CM-NWs, nickel ions were firstly reducted by a strong reduction agent of hydrazine hydrate and turned into tiny spherical particles owing to the aggregation effect (shown in Fig. 4a). Under an external magnetic field, the nanoparticles will be magnetized and self-assemble along the magnetic line of force, forming straight wires. The nanoparticles/nuclei in the solution will continually aggregate into the connection region of nanoparticles in the NWs according to the theory of minimized energy. It is indicated that the diameter of NWs increased, and the uniformity of NW’s diameter was improved. Moreover, some spiky Ni nanocrystallites distribute on the surface of NWs [15]. Figure 4b shows the distribution of CM-NWs after the experiment.

In the dropping method, because the density of the NiCl_2_·6H_2_O solution is larger than that of the reduction agent, the droplet was diffusing and falling in the reduction agent which is different from that of the conventional method. In Fig. 4c, the drop-shaped area bounded by the dotted line indicates the diffusion region of the NiCl_2_·6H_2_O solution in the reduction agent, and the diffusion region increased with the reaction time increasing. Therefore, Ni reduction reaction will follow the dropping process of droplet; the Ni-induced nanoparticles are a graded distribution. In addition, owing to the little Ni content in droplet, there are not enough nanoparticles to increase the diameter of NWs during the formation process, i.e., the diameter of DM-NWs is smaller, and the NWs have a smooth surface compared to the CM-NWs as shown in Fig. 2a, b. Meanwhile, inasmuch as the Ni nanoparticles were distributed in a small area, the magnetic induction line will concentrate upon this area, which will enlarge the length of NWs (shown in Fig. 4d).

**Fig. 5:**
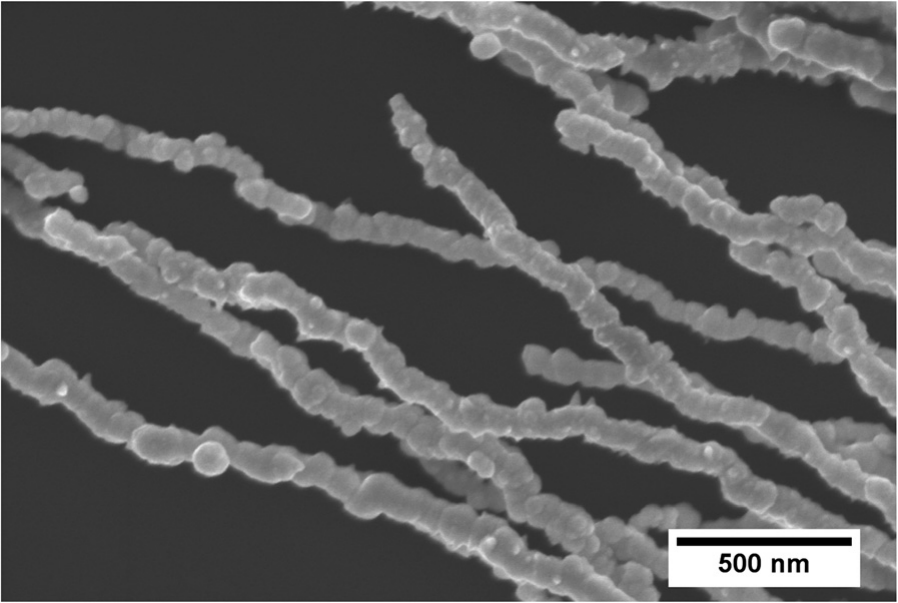
SEM image of NWs synthesized by the dropping method with the optimized process parameters. At the optimized process, the diameter of the NWs is ~70 nm and the length is ~45 μm

To synthesize the NWs, there are many factors, such as Ni concentration, temperature, and magnetic field strength, which influence the morphology of NWs [11]. By optimizing the synthesis parameters, we found that it is very difficult to synthesize the NWs with a diameter less than 300 nm using the conventional method [15]. However, the Ni NWs with a diameter and length of 70 nm and ~45 μm were synthesized successfully using the dropping method as shown in Fig. 5.

## Conclusions

In summary, a facile, low-cost, environment-friendly improved approach, dropping method, to synthesize Ni NWs with a high aspect ratio has been developed. By optimizing the preparation conditions, the diameter of NWs decreased to 70 nm using this method. Compared to the conventional method, it is indicated that the dropping method is an effective technique to tune the diameter of Ni NWs. Based on the investigation of the structures of NWs and the influence of synthesis parameters, the difference of the synthesized process between DM-NWs and CM-NWs has been discussed. This improved synthesis method shows great potential value to synthesized high-quality NWs which could be applied in the fields of catalytic, magnetic, and electric sensors.
